# Neuroendocrine Carcinoid Lung Tumor: A Case Series of an Indolent Tumor

**DOI:** 10.7759/cureus.72315

**Published:** 2024-10-24

**Authors:** Ratika Dogra, Kyle Schroeder, Krishna Chaudhary, Krishna Khatri, Vinod Khatri

**Affiliations:** 1 Internal Medicine, Bon Secours Mercy Health, Toledo, USA; 2 Pulmonary and Critical Care, University of Toledo, Ohio, USA; 3 Internal Medicine, Abington Memorial Hospital, Philidelphia, USA; 4 Internal Medicine, BJ Medical College, Ahmedabad, IND; 5 Pulmonary and Critical Care Medicine, St. Vincent Mercy Medical Center, Toledo, USA

**Keywords:** carcinoid tumor, chronic cough, dyspnea, hilar mass, neuroendocrine tumor

## Abstract

Neuroendocrine tumors (NETs) of the lung are infrequently encountered tumors, constituting a small subset of pulmonary neoplasms. The lung is the second most common site of carcinoid tumors. Most patients experience vague symptoms for many years, making them fairly difficult to diagnose. This case series presents three unique and challenging cases of pulmonary neuroendocrine tumors.

Case 1 is a 55-year-old female with a 6-month history of progressive dyspnea and noted to have a lung mass blocking the left main bronchus. The patient underwent bronchoscopy with tissue diagnosis and ultimately needed thoracotomy and left-sided pneumonectomy. Case 2 is a 61-year-old female with a chronic cough for several years who was seen to have a right lung mass, underwent a bronchoscopy showing a Carcinoid tumor, and was taken for a right lower lobe lobectomy. Case 3 is a 75-year-old female who was seen to have a right hilar mass as an incidental finding on CT imaging. The patient underwent bronchoscopy with the biopsy proving carcinoid tumor. The patient had multiple co-morbidities, was not deemed to be a surgical candidate, and was referred for radiation therapy.

The patients in the case series presented with nonspecific respiratory symptoms, leading to diagnostic investigations including imaging studies and bronchoscopy evaluation. Histopathological examination revealed a well-differentiated neuroendocrine tumor with positive immunohistochemical staining for neuroendocrine markers. Subsequent staging investigations confirmed localized disease without distant metastasis. Multidisciplinary collaboration involving pulmonologists, oncologists, radiation oncologists, and surgeons played a pivotal role in determining the optimal therapeutic approach. These patients underwent surgical resection, and postoperative pathology confirmed clear margins. Adjuvant therapy was considered based on the tumor characteristics and the overall risk of recurrence.

## Introduction

Neuroendocrine also called carcinoid tumors comprise around 2% of pulmonary neoplasms [1]. Carcinoids arise from neuroendocrine tumors arising from enterochromaffin cells. These are most common in gastrointestinal and bronchopulmonary systems. Most tumors run an indolent course, especially the ones that do not produce any hormones, and come to attention when the tumors reach a significant size for compressive symptoms. The lung is the second-most common site of carcinoid tumors. These tumors originate from the neuroendocrine cells that can produce amines and peptides. Approximately 30-40% of patients with well-differentiated neuroendocrine tumors present with carcinoid syndrome with the secretion of several humoral factors [2]. These tumors are generally rare and arise in the gastrointestinal tract most commonly. These tumors can be typical or atypical [3]. Typical carcinoids are slow-growing and usually do not spread beyond the source. Atypical carcinoids are faster growing and have more potential to spread to other organs [4]. The main histologic features for distinguishing typical and atypical carcinoid tumors are the morphology, mitotic count, and the presence or absence of necrosis. We present a case series of three cases with carcinoid lung tumors. Informed written consent was obtained from all three patients for the case series. The clinical presentation, diagnostic workup, and therapeutic management are detailed to provide insights into the intricacies of managing this rare entity. The three cases were interesting, as the patients did not present with any acute symptoms, and no history of weight loss was reported. Two of the patients presented with cough and gradual shortness of breath while the third patient was seen to have a lung mass as an incidental finding on CT imaging.

## Case presentation

Case 1

**Figure 1:**
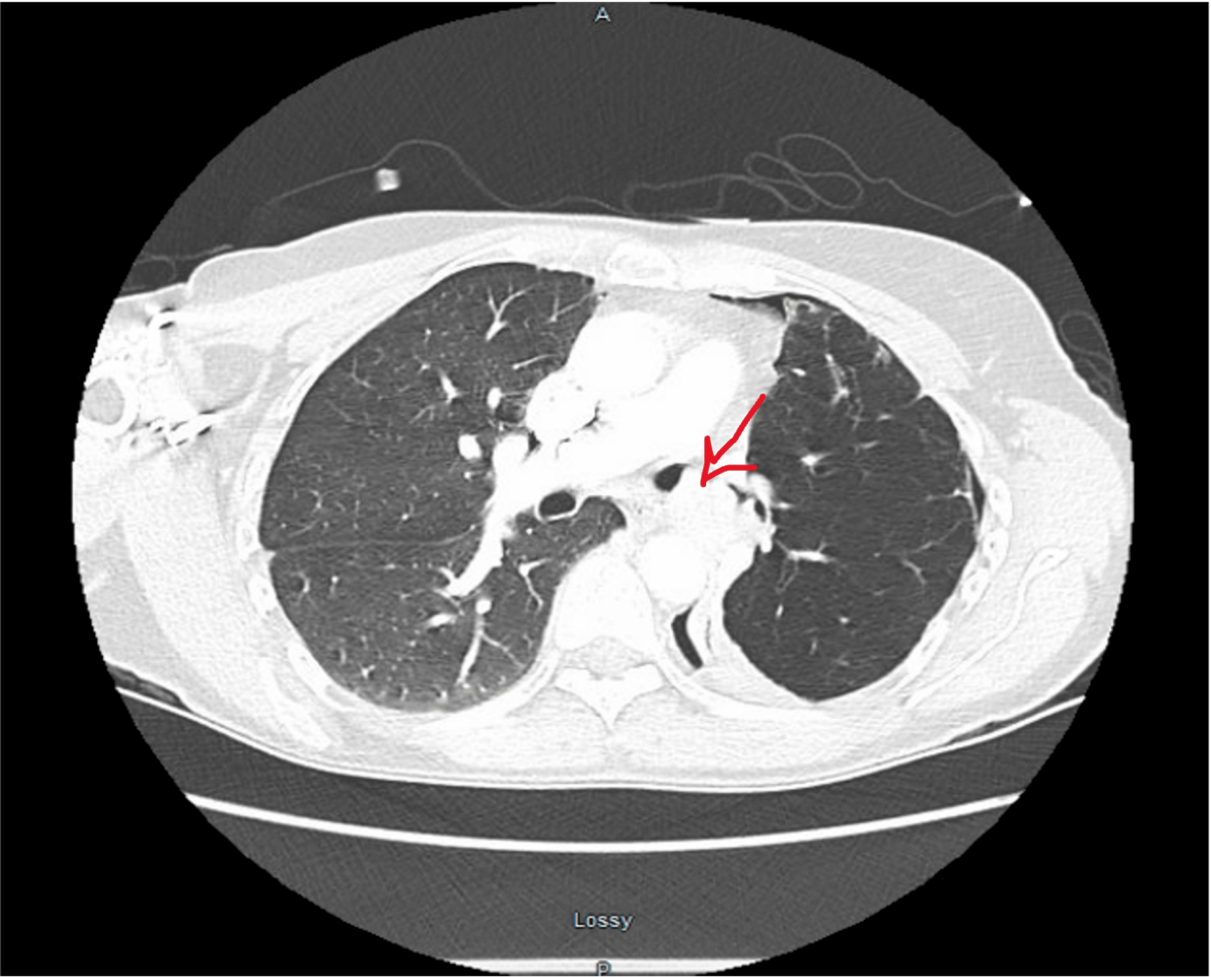
3.4 X 2.3 cm mass obstructing the left bronchus

A 55-year-old female, non-smoker, presented to the hospital with a cough for six months and progressive dyspnea on exertion. The patient initially had X-rays on an outpatient basis, which reported the possibility of chronic obstructive pulmonary disease (COPD) changes per the radiologist. Subsequent pulmonary function tests showed moderate ventilatory impairment. The patient denied any asthma and no occupational exposure. The patient was started on a trial of Advair. Her dyspnea was unresolved, and she had an episode of chest pain, which prompted her to go to the ER where a CT scan was concerning for a 3.4 X 2.3 cm mass obstructing the left bronchus (Figure 1). She underwent bronchoscopy and was noted to have complete obliteration of the left main bronchus by a large vascular mass. The patient was transferred to Cleveland Clinic where she had another bronchoscopy with a biopsy showing a carcinoid tumor. The patient was taken for VATS, which was converted to thoracotomy and left-sided pneumonectomy. The patient was monitored with surveillance.

Case 2

**Figure 2:**
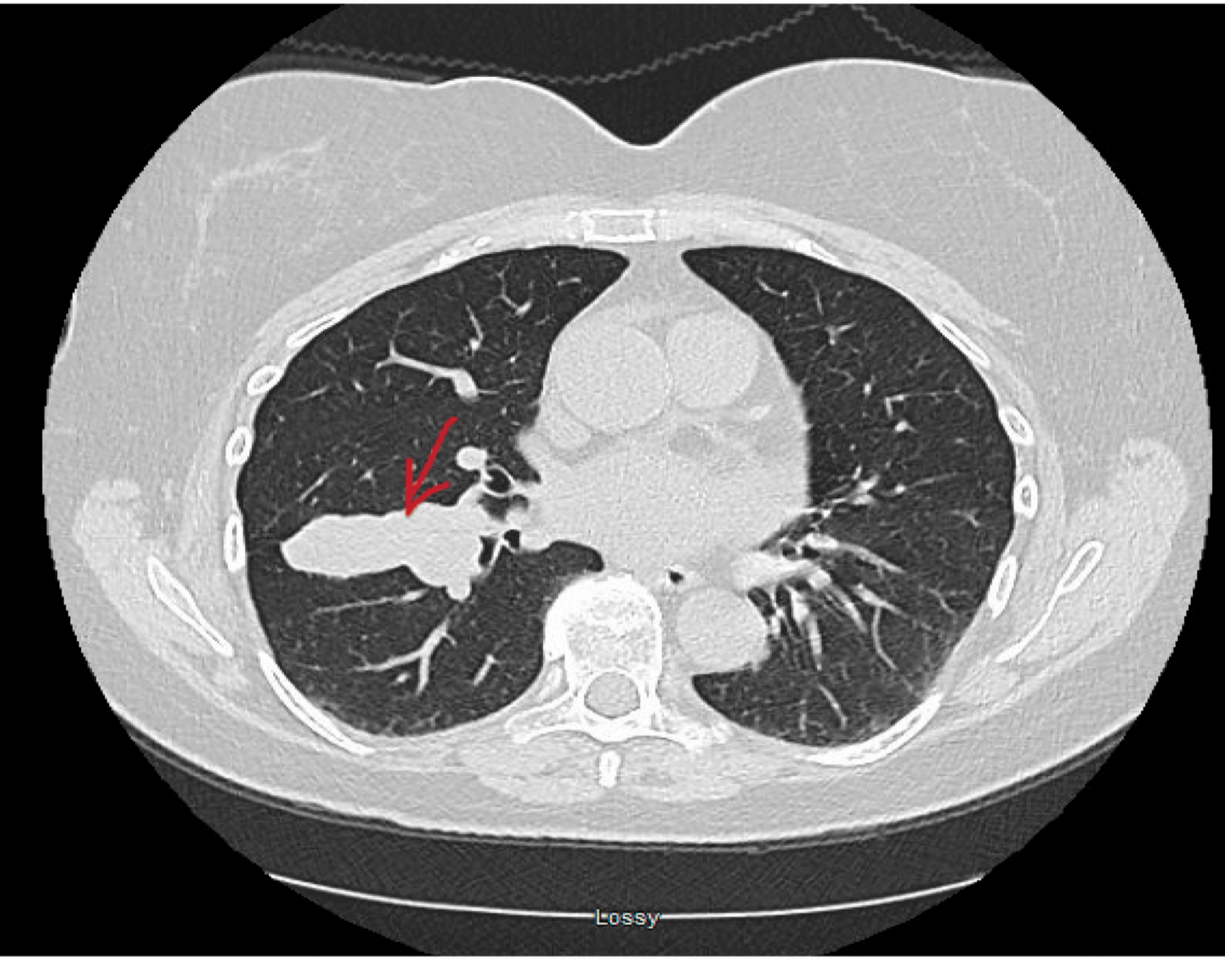
Lobulated mass in the anterior basal segment of the right lower lobe

A 61-year-old female, non-smoker, with a past medical history of hypertension presented with a chronic cough for several years associated with sputum production. The patient’s chest X-ray showed a right middle lobe infiltrate. It was seen that a CT scan four years ago showed concern for right-sided consolidation. Subsequent CT chest showed an ill-defined lobulated mass in the anterior basal segment of the right lower lobe (Figure 2). A review of the previous images showed that the lesion had increased in size. She underwent bronchoscopic evaluation, which showed a mass lesion in the anterior basal segment right lower lobe. The biopsy was consistent with a grade 1 carcinoid tumor. The patient was referred to CT surgery and a medical oncologist. The patient underwent a right lower lobectomy. Her surgical margins were reported to be clear, and there was no lymph node involvement.

Case 3

**Figure 3:**
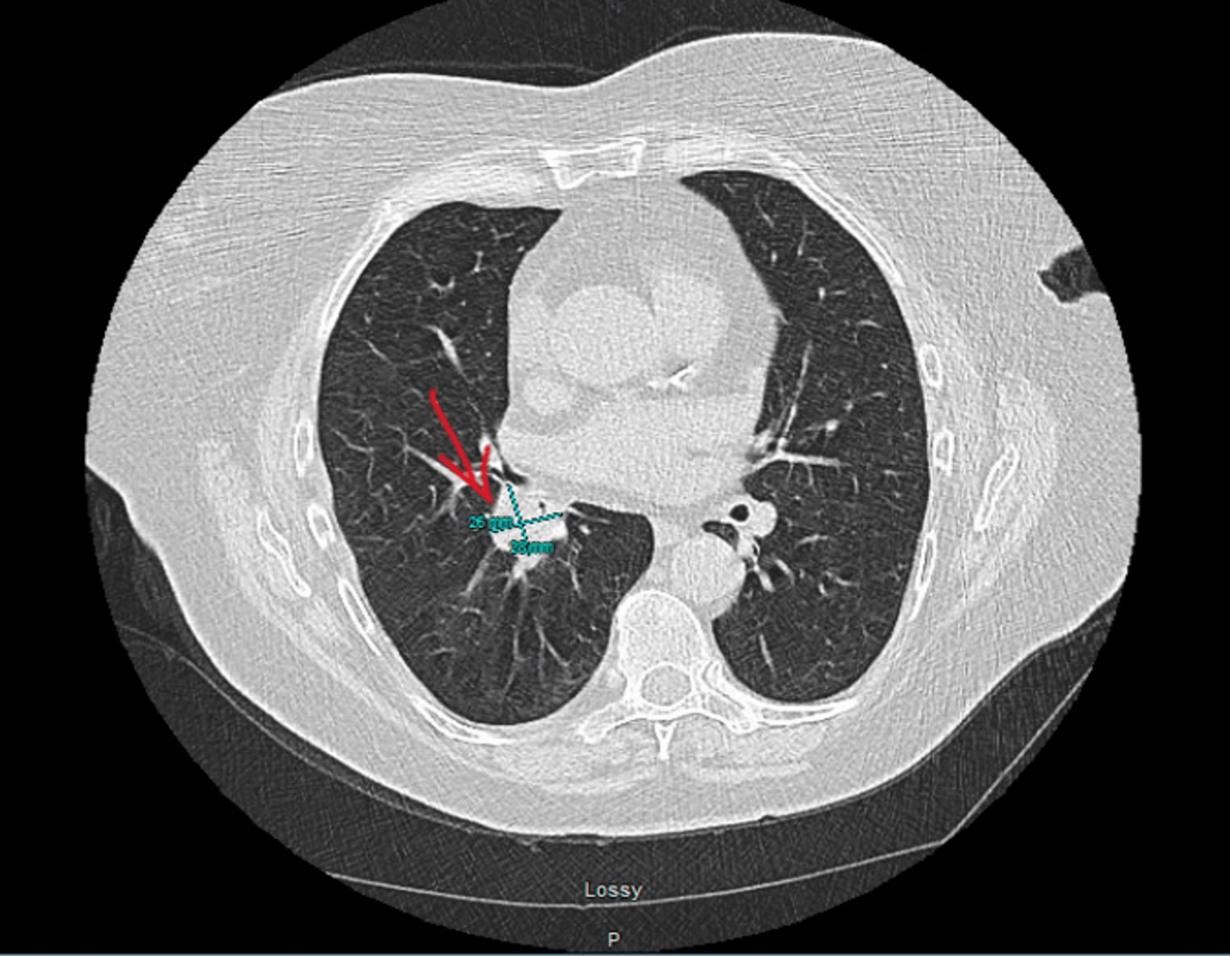
A right hilar mass measuring 2.6 X 2.8 cm causing a narrowing of the right lower lobe bronchus

**Figure 4:**
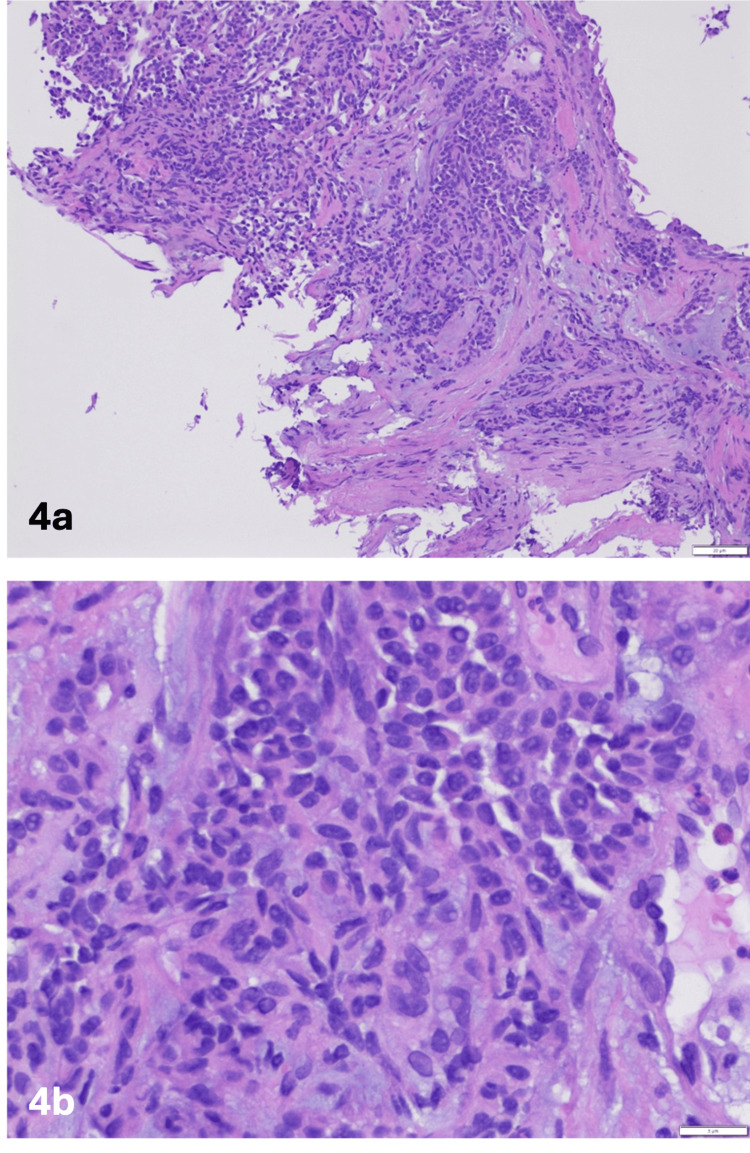
Histologic section showing uniform round cells with finely granular nuclear chromatin and abundant eosinophilic cytoplasm on H&E stain giving a salt and pepper appearance 4b is the magnified image of 4a.

**Figure 5:**
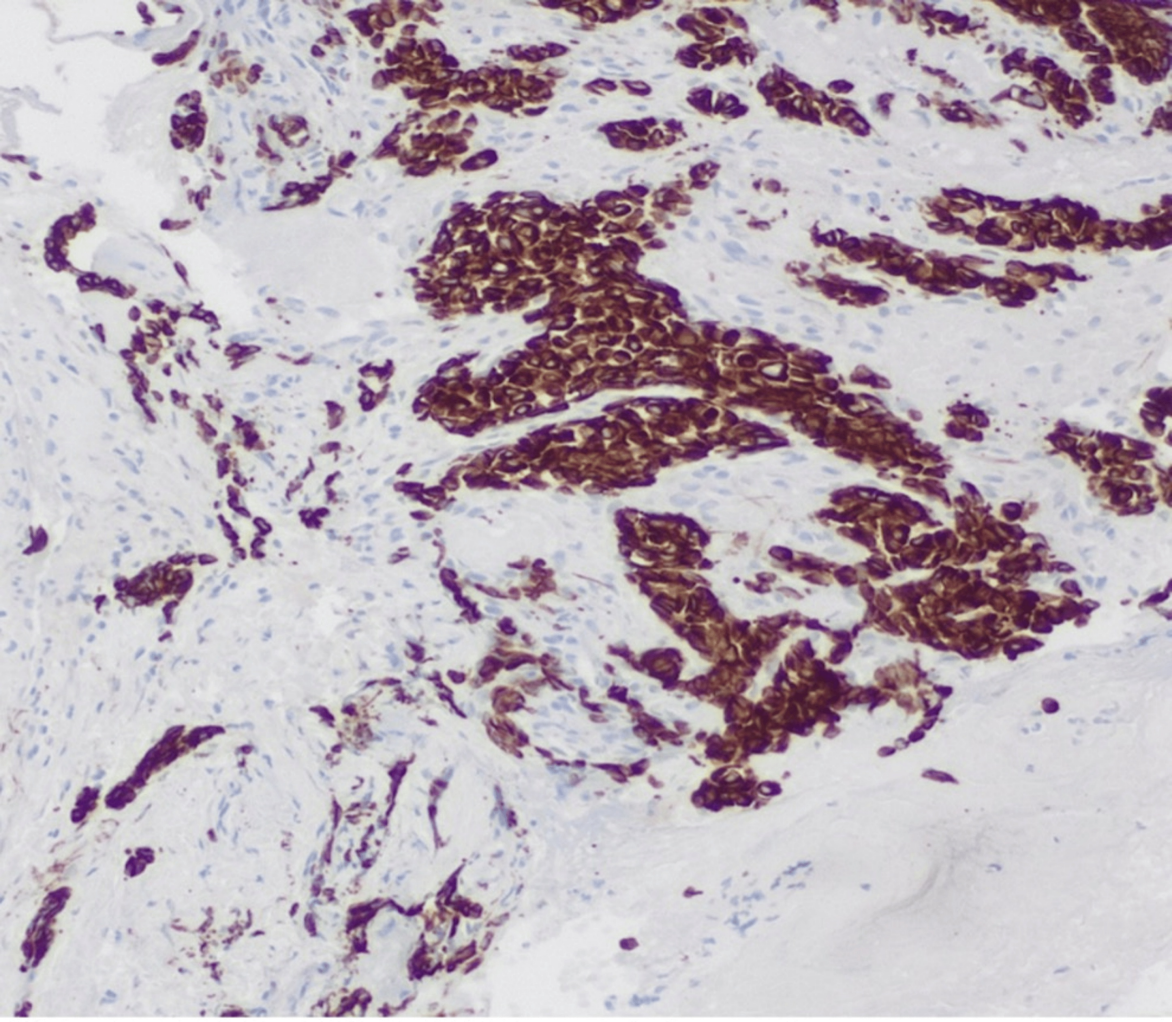
Carcinoid tumor with pan cytokeratin stain Carcinoids typically stain with low molecular weight cytokeratin antibodies, which are a combination of keratins.

A 75-year-old female with a past medical history of atrial fibrillation, on Xarelto, hyperlipidemia, hypertension, and obesity. The patient quit smoking 10 years ago in 2013. On a regular screening CT scan of the chest, there was a concern for the right hilar mass measuring 2.6X2.8 cm causing narrowing of the right lower lobe of the lung (RLL) bronchus (Figure 3). The patient underwent bronchoscopy with a right lower lobe biopsy showing a low-grade carcinoid tumor with a positive salt and pepper appearance on H&E stain and positive cytokeratin stain (Figures 4a, 4b, 5). The patient was referred to cardiothoracic surgery for pneumonectomy but due to multiple comorbidities and the patient’s age, the patient was recommended radiation therapy. The patient is awaiting a workup with a radiation oncologist.

## Discussion

Pulmonary neuroendocrine tumors are very rare epithelial cell malignancies, which are less than 1-2% of all lung cancers [1]. However, the lung is the second most common site of carcinoid tumors after the GI tract. There are two kinds of carcinoid tumors - typical and atypical, which are well-differentiated and less differentiated, respectively. Approximately 80% of tumors are central and 20% are peripheral. [5] These tumors are usually not associated with smoking. Due to the rarity, no clear staging system exists for these tumors. Although designed for non-small cell lung cancer, the tumor, node, metastasis (TNM) classification system has been applied to broncho-pulmonary carcinoids since 2010, and it is the approach that is currently recommended by both the European Neuroendocrine Tumor Society and North American Neuroendocrine Tumor Society [6,7].

Presentation

Most of the patients have very indolent symptoms. Lung carcinoids tend to be more common in the younger population as compared to other kinds of lung cancer. The average age of people when they are diagnosed is around 45 years for typical carcinoids and 55 years for atypical carcinoids [8,9]. Some patients complain of chronic cough over many years, some report exertional dyspnea, while others present with hemoptysis or wheezing [10]. Exertional dyspnea starts when the tumor becomes large enough to cause bronchial obstruction. A lot of times, these tumors are diagnosed on regular screening tests or diagnostic tests performed for other reasons. Sometimes symptoms are related to lung collapse or post-obstructive pneumonia distal to airway obstruction. Endocrine symptoms are fairly rare at the clinical level and thus are considered "endocrinologically silent," as they do not commonly produce significant amounts of hormones or bioactive substances that result in evident clinical effects. In cases where the carcinoid is active, the symptoms include face flushing, palpitations, elevated blood pressure, weight gain, and asthma-like symptoms.

Evaluation

Basic blood workups like blood counts, electrolytes, liver and kidney function along with plasma chromogranin A measurement are studies indicated in the diagnostic process and follow-up of carcinoids [11]. An X-ray can be useful in 40% of cases but a CT scan with contrast is the gold standard for radiological diagnosis. The CT features of peripheral carcinoid tumors presenting as solitary tumors, include lobulated nodules of high attenuation, nodules that densely enhance with contrast administration might have the presence of calcification, subsegmental airway involvement on thin-section analysis, and nodules associated with distal hyperlucency, bronchiectasis, or atelectasis [12]. Bronchoscopic evaluation is the gold standard for evaluation, as these tumors are located centrally. They do carry a bleeding risk due to high vascularity. Fluorodeoxyglucose (FDG) positron emission tomography (PET) may distinguish typical from atypical carcinoid tumors.

Pathophysiology and histology

Central carcinoid tumors are round and have well-defined boundaries. These display salt-and-pepper chromatin and have abundant eosinophilic cytoplasm. These tumors commonly obstruct the bronchial lumen. Generally, atypical carcinoids are more aggressive and larger [13]. Typical carcinoid tumors are characterized by having less than 2 mitoses per 2 mm² and do not show necrosis. In contrast, atypical carcinoids exhibit similar histological features as typical carcinoids but are defined by the presence of 2 to 10 mitoses per 2 mm² and tend to have necrosis as well [14]. Immuno-histochemistry has a crucial role in diagnosis when the sample size is small. A recommended antibody panel for immunostaining includes chromogranin A, synaptophysin, and CD56.

Treatment

All bronchial carcinoid tumors are malignant and do have the potential to metastasize [15]. Surgery is the best treatment of choice especially for atypical carcinoid tumors, as they are aggressive. Surgery is also advisable in cases of non-visualization of the distal and basal tumor parts. Management of carcinoid tumors of the bronchi has been done by endoscopic resection, sleeve lobectomy with parenchymal sparing, and pneumonectomy [16]. Laser and cryotherapy are being used for tumors with good bronchoscopy visualization of the distal and basal tumor margins with no evidence of bronchial wall involvement [16]. Hilar and mediastinal lymph node sampling and examination should be performed during open procedures.

## Conclusions

Our case series underscores the importance of a comprehensive diagnostic approach and highlights the complexities associated with the management of pulmonary neuroendocrine tumors. Most of these tumors do not exhibit endocrine function and have an indolent course. Presentation is due to the location of tumors compressing the pulmonary tissue. Exertional dyspnea starts when the tumor becomes large enough to cause bronchial obstruction. A lot of times, patients are seen to have the lung mass as an incidental finding on the imaging done for other reasons. Keeping the diagnosis in the differential is crucial for the evaluation of pulmonary tumors. The case series presents three cases with an indolent presentation of a lung tumor that remained hidden for years before diagnosis was made. Insights gained from this case series contribute to the growing body of literature on the clinical spectrum, diagnostic challenges, and therapeutic strategies for this rare subset of lung neoplasms. Further studies and long-term follow-up are essential to refine treatment paradigms and improve outcomes for patients with pulmonary neuroendocrine tumors.
